# Supplementary material for: LcTprxII Overexpression Enhances Physiological and Biochemical Effects in Maize Under Alkaline (Na2CO3) Stress
Source: Plants (Basel). 2025 May 14;14(10):1467. doi: 10.3390/plants14101467 (PMC12114990; doi:10.3390/plants14101467)
Supplement: Supplementary file 1 [file plants-14-01467-s001.zip › Supplementary Materials Images.pdf]

Figure S1 Multiple sequence alignment of LcTpxII

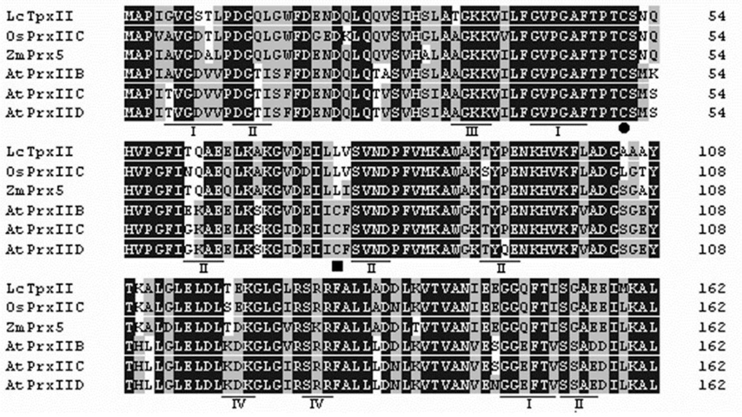

Figure S1 Multiple amino acid sequence alignment of LcTprxII and homologous genes and location of an endogenous gene on chromosome 6 (A) Multiple amino acid sequence alignment of *LcTprxII* and homologous genes in maize, rice, and Arabidopsis. At: *Arabidopsis thaliana*. The numbers on the right side correspond to actual amino acid numbers. LcTpxII: ACV20868; OsPrxIIC: Os01g48420; ZmPrx5-1: NP001149765; AtPrxIIB: AT1G65980; AtPrxIIC: AT1G65970; AtPrxIID: AT1G60740.

Figure S2 The expression pattern of the 10 genes identified by RNA-seq in transgenic maize

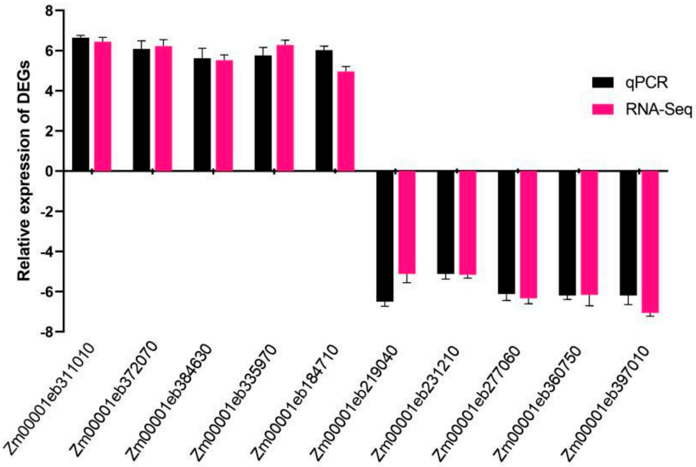

These 10 genes were randomly selected from the RNA-Seq data, which qRT-PCR verified. Table S2 illustrates the detailed list of genes in each group and the extent of changes.
